# Intraparenchymal meningioma mimicking cavernous malformation: a case report and review of the literature

**DOI:** 10.1186/1752-1947-8-467

**Published:** 2014-12-29

**Authors:** Senol Jadik, Alexandru C Stan, Uwe Dietrich, Terttu A Pietilä, Alaa Eldin Elsharkawy

**Affiliations:** 12 Department of Neurosurgery, University of Kiel, Arnold-Heller-Str.3, Haus 41, 24105 Kiel, Germany; 13 Department of Clinical Neuropathology, Evangelic Hospital Bethel, Remterweg 2, 33617 Bielefeld, Germany; 14 Department of Neuroradiology, University of Essen, Hufelandstraße 55, 45147 Essen, Germany; 15 Department of Neurosurgery, Evangelic Hospital Bethel, Burgsteig 13, 33617 Bielefeld, Germany

**Keywords:** Meningiomas, Intraparenchymal meningiomas, Cavernous malformation, Ossification, ‘popcorn’, Hemosiderin deposits

## Abstract

**Introduction:**

A primary intraparenchymal meningioma located in the subcortical region of the brain without a dural attachment is extremely rare. To the best of our knowledge, this is the first report showing that meningioma can mimic cavernous malformations.

**Case presentation:**

We present the case of a 42-year-old German man who presented to our institution with seizure. Both computed tomography and magnetic resonance imaging scans showed characters of an intra-axial subcortical lesion with a ‘popcorn’ appearance and hemosiderin deposits in the right parietal lobe. The initial diagnosis was cavernous malformation. Intraoperatively, the lesion presented as a subcortical mass that had no connection to the dura or the ventricle. The histological diagnosis showed a WHO Grade 1 ‘raddled’ psammomatous meningioma with extensive metaplastic ossification. A literature review of 29 cases of intraparenchymal meningiomas regarding their clinical presentations, location and management was performed.

**Conclusions:**

Meningiomas can be found in any region of the brain with and without dural attachment. Intraparenchymal meningiomas can have multiple entities mimicking their presentation. Caution must be used regarding the preoperative differential diagnosis.

## Introduction

Meningiomas account for approximately 15% of all intracranial neoplasms [1]. Meningiomas are usually attached to the dura and thought to arise from the arachnoid cap or meningothelial cells. Occasionally meningiomas develop without dural attachment, mainly in the intraventricular region, within the sylvian fissure, pineal region, or infratentorial compartment, and in pediatric and young patients [2–6]. The majority of meningiomas exhibit highly stereotypic imaging characteristics, which often facilitate their diagnosis without the need of invasive diagnostic procedures. In cases of absence of dural attachment, it is often difficult in the preoperative diagnosis to distinguish meningioma from high-grade glioma, cavernous angioma, or metastatic brain tumors and sarcomatous lesions [7–10]. Primary intraparenchymal meningiomas are rare and they are challenging to diagnose, especially when presenting with a typical magnetic resonance imaging (MRI) scan appearance.

We present a rare case of primary intraparenchymal meningioma that, preoperatively, was not typical for meningioma and showed characteristics of cavernous malformation. We reviewed all cases of intraparenchymal meningiomas in the literature to gather information regarding their sites, clinical presentations, pathological features, management, and outcome.

## Case presentation

**Figure 1:**
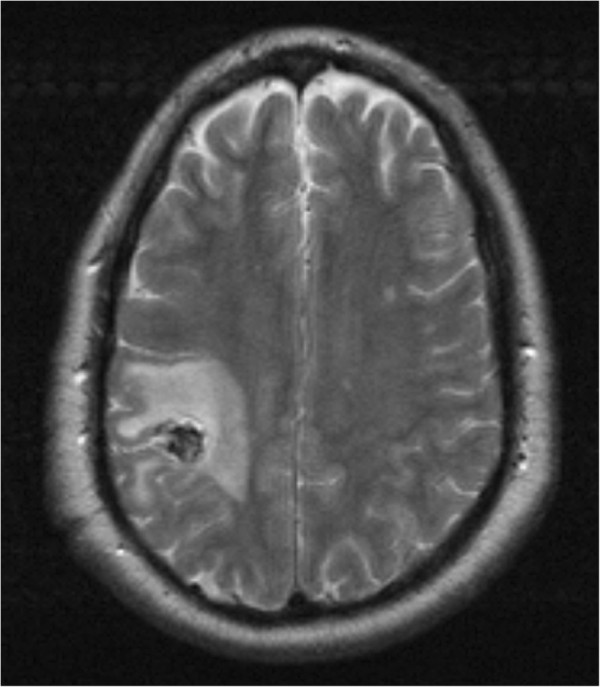
**Axial T2-weighted image demonstrating a popcorn-shaped predominantly hypointense lesion in the parietal lobe with perifocal edema.**

**Figure 2:**
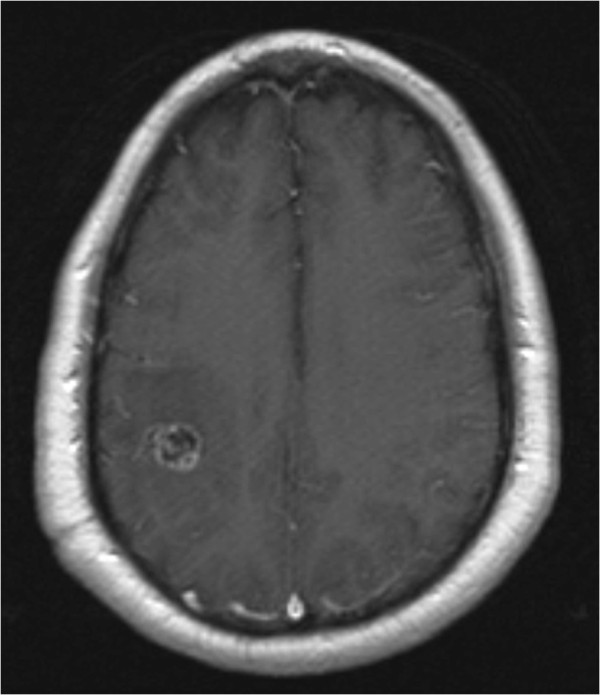
**Contrast-enhanced axial T1-weighted image revealing a heterogeneous contrast-enhancing lesion in the subcortical white matter of the right parietal lobe and no apparent dural attachment.**

**Figure 3:**
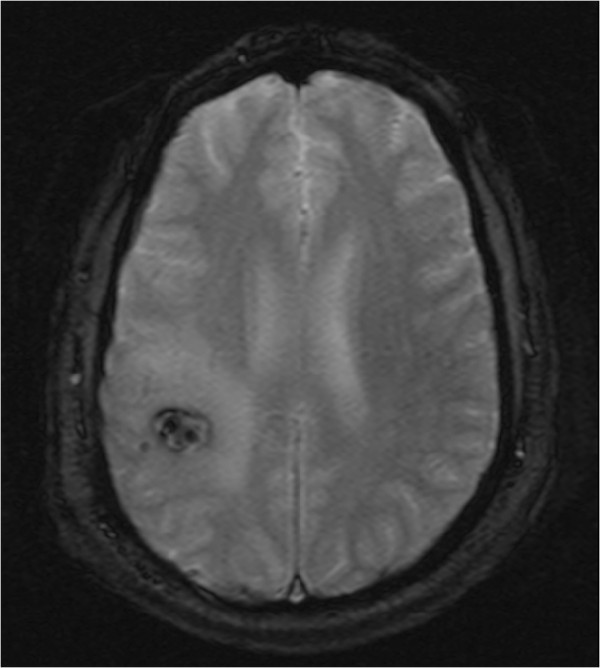
T2* gradient echo image showing a mass lesion with dots of calcification and surrounding hypointense rim typical of cerebral cavernoma.

A right-handed 42-year-old German man presented to our institution with convulsive seizures. There were no other neurological symptoms or signs and no neurological deficits. The initial computed tomography (CT) scan of his brain revealed a hyperdense lesion with calcification in the right parietal area. His cranial MRI scan (Figures 1, 2 and 3) demonstrated a popcorn-shaped mass in the subcortical white matter of his right parietal lobe. The lesion had smooth lobulated margins and no dural attachment was apparent. The lesion was hypointense on T1-weighted images and mixed hypo- and hyperintense on T2-weighted images. There were local mass effect, peritumoral edema and heterogeneously contrast enhancement. On his T2* gradient echo images the lesion showed focal spots of (dark signal) hypointense areas centrally compatible with calcifications, demonstrated on his CT scans, and a surrounding hypointense rim interpreted as hemosiderin deposition typical of cerebral cavernoma.

A right parietal craniotomy was performed under MRI-guided navigational assistance. Grossly, no mass or any other pathologic tissue was observed below the dura or on the cortex. Using a sulcal approach, the mass reached about 2cm into the cortex, showing a good cleavage. The mass was completely calcified (like a stone) and measured about 1.5×1.5cm in diameter. A gross total resection could be achieved. Postoperatively, he had no neurological deficits.

**Figure 4:**
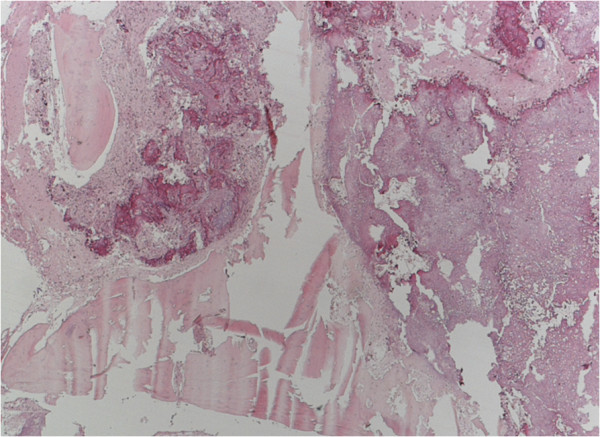
**Routine hematoxylin and eosin histology revealed a tightly packed and parallel laminated mass of fibrous tissue (4× objectives).**

**Figure 5:**
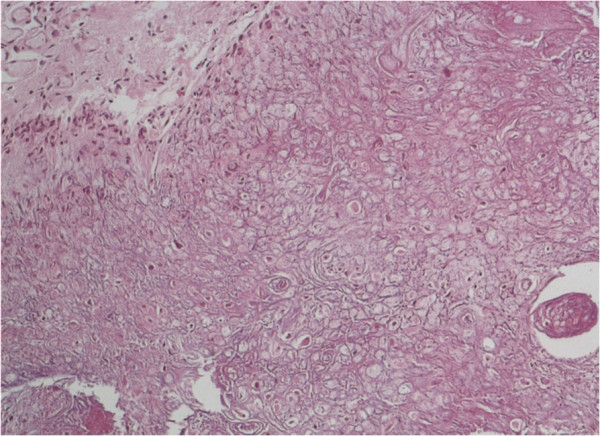
**Routine hematoxylin and eosin histology revealed many faded whorl structures and psammoma bodies (10× and 20× objective).**

A histological examination of the specimen showed (at low magnification (5× objectives)) a tightly packed and parallel laminated mass of fibrous tissue (Figure 4). There were a few irregularly shaped small foci within the lesion, which were partly filled with flimsy tissue. At higher magnification (10× and 20× objective), many faded whorl structures and psammoma bodies were identified (Figure 5).

**Figure 6:**
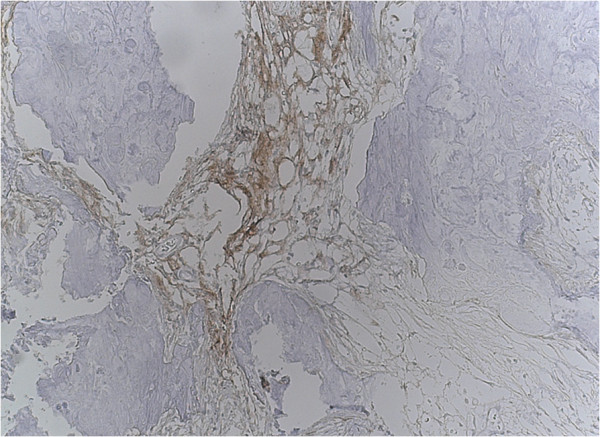
**Immunohistochemistry analysis revealed focal positivity for endothelial membrane antigen (20× objective) and psammoma bodies (20× objective).**

Furthermore, the lesion contained areas of transition into mature lamellar bone and was partly bordered by brain tissue with piloid gliosis. There was no periodic acid-Schiff-positive inclusions, nor hemosiderin deposits. An immunohistochemistry analysis revealed focal positivity for epithelial membrane antigen and Vimentin (Figure 6). Staining the sample with reticulin did not reveal a hemangiopericytomas-like pattern. No other histological sign of atypia or malignancy was observed. The pathological examination was performed by two independent and board-certified pathologists. The final diagnosis was a ‘raddled’ psammomatous meningioma with extensive metaplastic ossification. The postoperative course was uneventful. He was discharged and returned to everyday life, showing no deficits.

In the literature review, we identified 36 cases of intraparenchymal meningiomas, including our case report. Case reports with insufficient information and meningiomas secondary to underlying meningioangiomatosis were excluded; 29 cases were included in total. There were 18 male (62.1%) and 11 female (37.9%) subjects, with the age ranging between 0.4 and 60 years (mean: 21.2 years). Of the 29 intraparenchymal meningioma cases, 26 were located in the supratentorial region (89.7%) and three were located in the infratentorial region of the brain (10.3%). The frontal location was dominant, being reported in 12 cases (41.4%). The most frequently reported clinical presentation was seizure, being reported in 20 cases (69.0%). In almost all of the cases, the radiological appearance was a solid mass showing contrast enhancement.

Preoperatively, three cases mimicked a glioma, one case mimicked metastasis und one case mimicked a cavernoma. Total surgical resection was the standard treatment approach and was achieved in all the cases, except in three cases involving the brainstem, in which only a subtotal resection was performed. Radiotherapy following the surgery was done in four cases und one subject additionally underwent adjuvant chemotherapy. Regarding the histopathology, the fibrous type proved to be dominant, being reported in 11 cases (37.9%). The majority of the patients had no postoperative deficits. The location, clinical presentation, radiological finding, and outcomes are summarized in Table 1.

**Table 1:** **All intraparenchymal meningiomas until January 2014**

Series	Gender/age (years)	Location	Clinical presentation	Radiology	Surgery	Pathology	Post-operative treatment	Recurrence	Outcome follow-up (years)
Emoto, 1954 [11]	F/26	Frontal	Motor disorder	Solid mass/no CM	GTE	fibrous	NS	NS	NS
Drake, 1986 [12]	M/12	Temporal	Seizure	NS	STE	Transitional	NAT	NS	3
Suematsu, 1974 [13]	M/0.4	Parietal	Seizure	NS	GTE	Fibrous	NS	No	2.5
Morimoto, 1976 [14]	F/17	Parietal	Seizure macrocephaly	Solid mass/no CM	GTE	Anaplastic	NS	No	2.4
Mena 1978 [15]	31/M	Temporo-parietal	Seizure	Solid mass	GTE	Sarcomatous	NS	No	0.8
Legius, 1985 [16]	M/1.2	Parietal	Seizure	hyperdense/CM only CT	GTE	Fibrous	NAT	No	ND/2.2
Schroeder, 1987 [17]	M/7	Frontal	Seizure	Classified mass/no CM	GTE	Fibroblastic	NS	NS	NS
Kimura 1987 [18]	M/0.9	Frontal	Seizure	NS	GTE	Fibrous	NAT	No	ND/5
Sakaki 1987 [19]	M/0.9	Frontal	Seizure	Solid mass/homogeneous CM	GTE	Fibrous	NAT	No	Good/5
Mamourian, 1991 [20]	F/2	Frontal	Macrocephaly	NS	GTE	Psammomatous	NS	No	0.3
Matsumoto 1992 [21]	M/6	Frontal	Seizure	lesion/CM	GTE	Transitional	NS	NS	2
Nakahara, 1993 [22]	M/56	Cerebellar cortex	No	Round mass/partial CM	GTE	Fibroblastic	NAT	No	ND
Kaneko, 1993 [23]	F/12	Temporal	Seizure	Solid mass/CM	GTE	Meningothelial	NS	NS	NS
Kohama, 1996 [24]	F/1.8	Frontal	Seizure	Solid mass/CM	GTE	Fibroblastic	NAT	No	ND/2
Sathi S, 1992 [25]	M/23	Frontal	Seizure	Smooth lobulated mass/no CM	GTE	Psammomatous	NAT	No	ND
Sanli, 1996 [26]	M/23	Frontal	Seizure	Low intense mass	GTE	Psammomatous	NS	NS	NS
Starshak, 1996 [27]	M/6.8	Frontal	Headache	Cyst/heterogeneous CM	GTE	Sarcomatous	Rd, ch	No	5
Teo 1998 [28]	F/1.8	Brainstem	Hemiparesis	Multi-lobulated mass + CM	STE	Clear cell	Rd refused	70% remnant	Poor
Shimizu, 1999 [29]	F/46	Parietal	Seizure	Low intense	GTE	Fibrous	NAT	No	NS
Wada, 2000 [10]	F/45	Parietal	Sensible disorders seizure	Solid mass + cyst/homogeneous CM (glioma)	GTE	Chordoid	NAT	No	ND/0.8
Karadereler, 2004 [2]	M/14	Temporal	Seizure	Solid mass/heterogeneous CM	GTE	Fibrous	NS	No	ND/3
Tekko, 2005 [30]	F/54	Temporal	Headache	Solid mass/homogenous CM (metastatic)	GTE	Fibrous	NAT	No	ND/1
Kim, 2005 [31]	F/41	Frontal	Seizure	Solid mass + cyst + CM (glioma, metastatic, meningioma)	GTE	Anaplastic	NAT	No	ND/1.3
Zhang, 2007 [32]	M/16	Occipital	Seizure	Solid mass + cyst + CM	GTE	Atypical	Rd	No	ND/1.6
Dutta, 2009 [33]	F/36	Temporal	Headache	Solid mass + cyst + CM	GTE	Rhabdoid	Rd	Yes	Poor
Yamada, 2010 [34]	M/60	Parietal	Motor weakness	Solid mass + cyst + CM (glioma)	STE	Meningothelial	NAT	Rest	ND
Shimbo, 2011 [35]	M/10	Frontal	Seizure	Solid mass + CM edema	GTE	Meningothelial with chordoid	NAT	No	ND/0.4
Jiang, 2012 [36]	M/23	Brainstem	Motor weakness and facial palsy	Solid mass + cyst + CM	GTE	Papillary WHO III	Rd	Yes	ND
Present case	M/42	Parietal	Seizure	Solid mass + CM (cavernoma)	GTE	Psammomatous	NAT	No	ND/2

Ch: chemotherapy; CM: contrast enhancement; CT: computed tomography scan; F: female; GTE: gross total excision; M: male; NAT: no adjuvant therapy; ND: no deficits; NS: not stated; Rd: radiotherapy; STE: subtotal excision; WHO:World Health Organization [37].

## Discussion

Intraparenchymal meningiomas are meningiomas that arise within the brain tissue without dural attachment and have also been used to characterize meningiomas that are not dural based [10]. The etiology of intraparenchymal meningiomas is unclear. Some authors presume that intraparenchymal meningiomas arise from arachnoid cells of the pia mater, which enter the surface of brain or sulcus with perforating blood vessels. Others suggest that the arachnoid cell rests during the migration progress [10]. The pineal region, the intraventricular region, and within the sylvian fissure are typical locations where a meningioma may develop without dural attachment [5, 38–40]. Meningiomas show characteristic imaging features such as: broad-based dural attachment, signal changes in the skull due to tumor infiltration, sharp demarcation between the tumor and the brain, mass effect on adjacent brain tissue, and homogeneous enhancement of a contrast agent [41, 42]. The site of origin provides a clear diagnosis in most cases. However, meningiomas can be mimicked by other intracranial tumors and pseudo-tumors such as glioma, ependymoma, metastasis, and cavernous malformation [7, 43].

Our case report shows several factors which pointed to the diagnosis of cavernous malformation: the subcortical location, clinical presentation, radiological findings like the characteristic popcorn appearance on the MRI scan, and hemosiderin deposition. Other findings, such as peritumoral edema, calcifications, hypointense appearance to gray matter on T1-weighted MRI scans, and mixed signal intensity on T2-weighted MRI scans, are also reported in cavernous malformations in different stages after hemorrhage. It has been reported that cavernoma malformations may mimic meningioma [25, 44–46]. Cavernous malformations may be misdiagnosed as the more commonly seen meningiomas, particularly when they do not display the same MRI characteristics as a typical cavernoma [45, 17]. To the best of our knowledge, this is the first report demonstrating that meningiomas can mimic cavernous malformations.

Our case report highlights the fact that preoperative diagnosis of atypical meningioma is challenging and classical MRI features may not be sufficient to distinguish the different pathologic entities, moreover, they can be misleading. Therefore, caution must be used in the preoperative differential diagnosis. In our case report, an angiography could have helped to distinguish the meningioma from a cavernous malformation, but this is not guaranteed due to the possibility of showing the same radiological features [41].

The literature review shows that intraparenchymal meningiomas occur in all regions of the brain, including supratentorial and infratentorial areas. Intraparenchymal meningiomas were, in most cases, not considered and misdiagnosed as other lesions. The clinical presentation was site dependent; seizures were the most common symptom. Unlike the usual meningioma, where the meningothelial variant of meningioma is the most common, the fibrous variant of meningioma is dominant in intraparenchymal meningiomas [47]. It seems that the fibrous variant of meningioma is generally dominant in meningiomas without dural attachment, such as intraventricular meningiomas. The large number of published cases of meningiomas without dural-base attachment establishes the fact that not all meningiomas are dural based.

## Conclusions

The meningioma in our patient had two unusual features: the subcortical location and the radiological appearance mimicking cavernous malformation. This case report highlights the fact that the typical radiological appearance of one lesion may sometimes be misleading. Intraparenchymal meningiomas can have multiple entities mimicking their presentation. Caution must be used in the preoperative differential diagnosis.

## Consent

Written informed consent was obtained from the patient for publication of this case report and any accompanying images. A copy of the written consent is available for review by the Editor-in-Chief of this journal.

## Abbreviations

CT: Computed tomography
MRI: Magnetic resonance imaging.
